# Childhood Obesity, MMP-9 Levels, and Vitamin D

**DOI:** 10.5935/abc.20170127

**Published:** 2017-10

**Authors:** Zeynep Cerit

**Affiliations:** Near East University Hospital, Nicosia - Chipre

**Keywords:** Pediatric Obesity, Child, Adolescents, Cardiovascular Diseases, Overweight, Matrix Metaloproteinase 9

**Dear Editor,**

I have read the article entitled “MMP-9 Levels and IMT of Carotid Arteries are Elevated in Obese Children and Adolescents Compared to Non-Obese” by Andrade et al.^1^, recently published in journal, with great interest. The investigators reported that obese children and adolescents present higher mean intima-media thickness (IMT), plasma matrix metalloproteinase (MMP)-9 and MMP-9/tissue inhibitor of metalloproteinase-1 ratio compared to the non-obese. Thus, these findings indicate that this group presents a risk profile for early atherosclerosis.^1^

Childhood obesity is an international public health problem leading to an increased risk of adult obesity and associated with cardiovascular diseases such as hypertension, peripheral and coronary artery disease.^2^ Vitamin D (vit D) may regulate adipose tissue mass, differentiation, and metabolism. Vit D deficiency might contribute with overweight and/or obesity, possibly by effects on lipogenesis and/or adipogenesis.^3^ Coussens et al.^4^ reported an inverse correlation between circulating vit D concentration and serum inflammatory biomarkers. Increased tumor necrosis factor-alpha (TNF-α) is associated with low vit D concentrations. Vit D down-regulates MMP-9 production by TNF-α and decreases production of MMP-9. Wang et al.^5^ reported that vit D derivatives could significantly inhibit TNF-α induced MMP-2 and MMP-9 secretion in nasal polyp-derived fibroblasts.

In this context, considering a close association among childhood obesity, serum MMP-9 and vit D levels, the correlation of this study’s result^1^ with serum vit D levels might be beneficial.
