# Supplementary material for: Childhood Obesity, MMP-9 Levels, and Vitamin D
Source: Arq Bras Cardiol. 2017 Oct;109(4):380–1. doi: 10.5935/abc.20170127 (PMC5644221; doi:10.5935/abc.20170127)
Supplement: Related texts

# Reply

**Authors:** Claudio Andrade, Adriana Bosco, Valeria Sandrim, Francisco Silva

We would like to thank you and agree with the considerations inserted in the letter about obesity and atherosclerosis. Indeed, we found a close association between obese children and adolescents with atherosclerosis, compared to non-obese ones.^1^

Besides, many authors have cited the important role of Vitamin D in these situations. Certainly, there is an inverse association between Vitamin and obesity. In this point, the Vitamin should regulate the adipose tissue metabolism, although its real role is still unknown, that is, whether it is cause or consequence.^2^

Indeed, obesity and Vitamin D deficiency represent an important health concern in the United States among children and adults.^2^

Because of the association between cardiovascular risk in obese children and adolescents, Atabeck et al.^3^ suggest the prescription of Vitamin D as a way to prevent the premature onset of atherosclerosis. Gul at al.^4^ reported that Vitamin D deficiency could contribute with morbidities associated to childhood obesity, such as increasing cardiovascular cardiometabolic risks, atherogenic dyslipidemia and hypertension.

In short, we think it would be important to promote more studies in order to prevent and treat atherosclerosis in obesity, as well to considerer the other diseases.

Sincerely,

**Claudio Andrade** **Adriana
Bosco** **Valeria Sandrim** **Francisco
Silva**

## References

[1] Andrade C, Bosco A, Sandrim V, Silva F (2017). MMP-9 levels and IMT of Carotid Arteries are elevated in
Obese Children and Adolescents Compared to Non-Obese. Arq Bras Cardiol.

[2] Peterson CA, Belenchia AM (2014). Vitamin D deficiency and childhood obesity: a tale of two
epidemics. Mo Med.

[3] Atabeck ME, Eklioglu BS, Akyürek N, Alp H (2014). Association between vitamin D level and cardiovascular risk
in obese children and adolescents. J Pediatr Endocr Met.

[4] Gul A, Ozer S, Yılmaz R, Sonmezgoz E, Kasap T, Takçı S (2017). Association between vitamin D levels and cardiovascular risk
factors in obese children and adolescents. Nutr Hosp.
